# First demonstration of multi-color 3-D *in vivo* imaging using ultra-compact Compton camera

**DOI:** 10.1038/s41598-017-02377-w

**Published:** 2017-05-18

**Authors:** Aya Kishimoto, Jun Kataoka, Takanori Taya, Leo Tagawa, Saku Mochizuki, Shinji Ohsuka, Yuto Nagao, Keisuke Kurita, Mitsutaka Yamaguchi, Naoki Kawachi, Keiko Matsunaga, Hayato Ikeda, Eku Shimosegawa, Jun Hatazawa

**Affiliations:** 1 Waseda University, Graduate School of Advanced Science and Engineering, Tokyo, Japan; 2 Hamamatsu Photonics K. K., Central Research Laboratory, Sizuoka, Japan; 3 National Institutes for Quantum and Radiological Science and Technology, Gunma, Japan; 4 Osaka University Graduate School of Medicine, Medical Imaging Center for Translational Research, Osaka, Japan

## Abstract

In the field of nuclear medicine, single photon emission tomography and positron emission tomography are the two most common techniques in molecular imaging, but the available radioactive tracers have been limited either by energy range or difficulties in production and delivery. Thus, the use of a Compton camera, which features gamma-ray imaging of arbitrary energies from a few hundred keV to more than MeV, is eagerly awaited along with potential new tracers which have never been used in current modalities. In this paper, we developed an ultra-compact Compton camera that weighs only 580 g. The camera consists of fine-pixelized Ce-doped Gd_3_Al_2_Ga_3_O_12_ scintillators coupled with multi-pixel photon counter arrays. We first investigated the 3-D imaging capability of our camera system for a diffuse source of a planar geometry, and then conducted small animal imaging as pre-clinical evaluation. For the first time, we successfully carried out the 3-D color imaging of a live mouse in just 2 h. By using tri-color gamma-ray fusion images, we confirmed that ^131^I, ^85^Sr, and ^65^Zn can be new tracers that concentrate in each target organ.

## Introduction

The devices that visualize *in vivo* radiation distribution play an important role in the field of nuclear medicine. Single photon emission tomography (SPECT) and positron emission tomography (PET) have been widely used for diagnosis at the early stage of desease and other medical conditions. Although these modalities have achieved valuable results, the radioactive tracers suitable for each detector are limited in terms of energy: SPECT only images low-energy gamma rays less than 400 keV and PET can image only positron emitting sources that emit 511 keV gamma rays. A Compton camera^1–3^, which estimates the direction of incident gamma rays based on Compton kinematics, features a wide energy range from a few hundred keV to several MeV. Hence, the Compton camera is promising detector that makes it possible to utilize a wider range of radionuclides that were never available before. Furthermore, a wider energy range enables the simultaneous imaging of multiple tracers, which provide not only more detailed information on lesions, but also identifies the element that is relevant to a specific function at once^4, 5^. For example, diagnoses such as observing graft survival and distinguishing between cancer and inflammation are made possible by utilizing information from multiple tracers. In addition, extending the types of available radioisotope (RI) tracers has the potential to reduce the costs of tracer production, which is usually made by the cyclotron facility in each medical center. Although various studies have been conducted for this application^6–11^, Compton cameras have never seen practical applications in the field of nuclear medicine. The main problems are (1) its low detection sensitivity compared to other conventional modalities such as PET and SPECT, and (2) the complexity of image reconstruction which arises from utilizing Compton kinematics.

In our previous study, we developed a Compton camera aimed at achieving high detection efficiency and practical spatial resolution. The camera consists of Ce-doped Gd_3_Al_2_Ga_3_O_12_^12, 13^ and multi-pixel photon counter arrays in both the scatterer and absorber^14–18^. The medical Compton camera is compact (4.9 cm × 5.6 cm × 10.6 cm) and light (580 g). Hence, it enables flexible measurement depending on the situation. The camera has a spatial resolution better than 3 mm (full width at half maximum, FWHM) and an intrinsic detection efficiency of 0.06% for 662 keV gamma rays^18^.

In this study, we demonstrated the feasibility of new *in vivo* imaging using our Compton camera. While there have been many other research studies adopting a Compton camera for nuclear imaging, they have not been able to provide a 3-D isotropic image in a real sense, owing to the vast bulk of the detector system. Moreover, each measurement took a very long time owing to the low sensitivity of detectors to gamma rays. In this paper, we attempt to resolve these problems by adopting multi-angle data acquisition using an ultra-compact Compton camera that also features high sensitivity. In particular, we provide for the first time 3-D isotropic imaging of a live mouse, which is detailed below. First, we tested experimental 3-D imaging performance using a ^22^Na point source and an uniform plane source of ^137^Cs. Based on this investigation, we conducted 3-D imaging of a live mouse with multiple radioactive tracers.

## Results and Discussion

### 3-D imaging of point source

**Figure 1:**
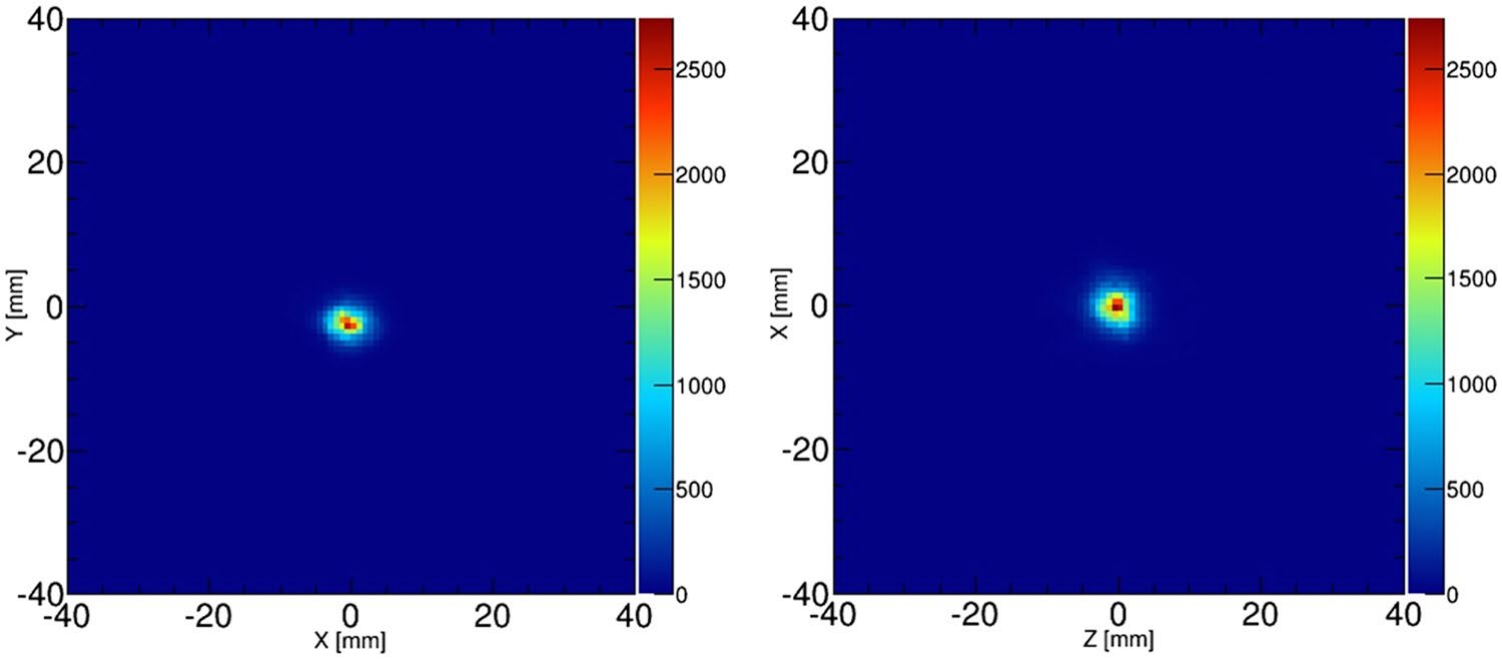
2-D slices of the ^22^Na point source image in the X-Y plane (*left*) and Z-X plane (*right*).

First, we evaluated the spatial resolution of our Compton camera by the measurement of a point source of ^22^Na in 3-D space. Figure 1 shows 2-D slices of reconstructed 3-D maximum likelihood expectation maximization (MLEM) images of the ^22^Na source at the center of the field of view after 15 iterations. The spatial resolutions which were obtained by FWHM of 1-D profiles were 3.19 mm, 2.76 mm, and 3.51 mm in the X, Y, and Z directions, respectively. These values are considerably consistent with the intrinsic angular resolution as reported in our previous work^18^.

### 3-D imaging of uniform plane source

**Figure 2:**
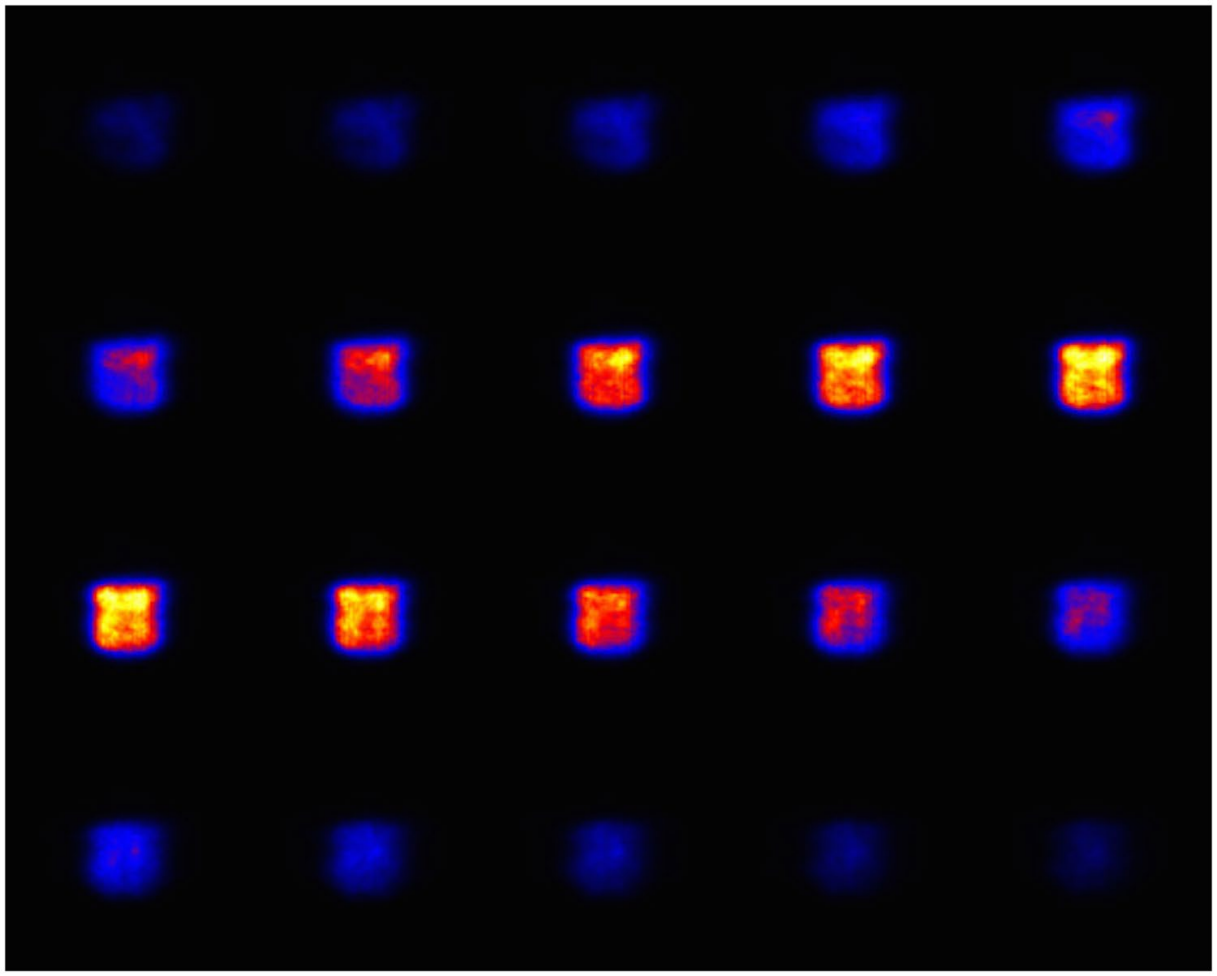
2-D slices of the plane source image. Each figure shows 0.8 mm pitch slice in the Z-Y plane.

**Figure 3:**
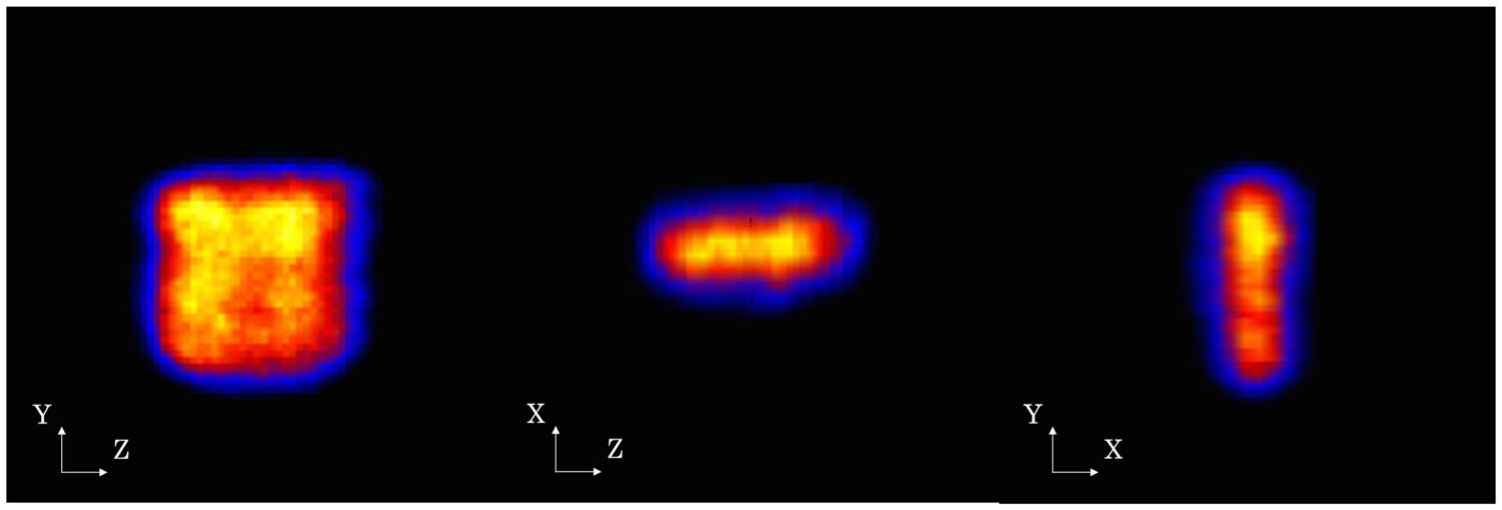
2-D slices of the 3-D imaging result at the center. (*Left*) in the Z-Y plane, (*center*) in the Z-X plane, and (*right*) in the X-Y plane.

In order to evaluate the imaging performance of the medical Compton camera and image reconstruction method, we first conducted an imaging experiment with a diffuse source of ^137^Cs. The total detected number of events used for reconstuction was 1.12 × 10^5^ events. Figure 2 shows 2-D slices of reconstructed 3-D MLEM images after 30 iterations. Each figure shows a 0.8 mm pitch slice in the Z-Y plane. Figure 3 (*left*), (*center*), and (*right*) show the center slice of the plane source in the Z-Y, Z-X, and X-Y planes, respectively. With regard to the typical value of the spatial resolution, the edge delineation in the center slice in the X, Y, and Z directions were comparable to 5.90 mm (FWHM), 5.33 mm (FWHM), and 6.31 mm (FWHM), respectively. Furthermore, the image intensity uniformity inside the region of interest (ROI), which was defined except for the source edge, was 11.5% by 1*σ*. On the basis of these results, we can verify the 3-D configuration of the square source. These imaging results suggest the promising performance of our Compton camera and image reconstruction method for 3-D imaging, even for the diffuse source with a uniformity accuracy of approximately 10%.

### 3-D *in vivo* imaging with a living mouse

**Figure 4:**
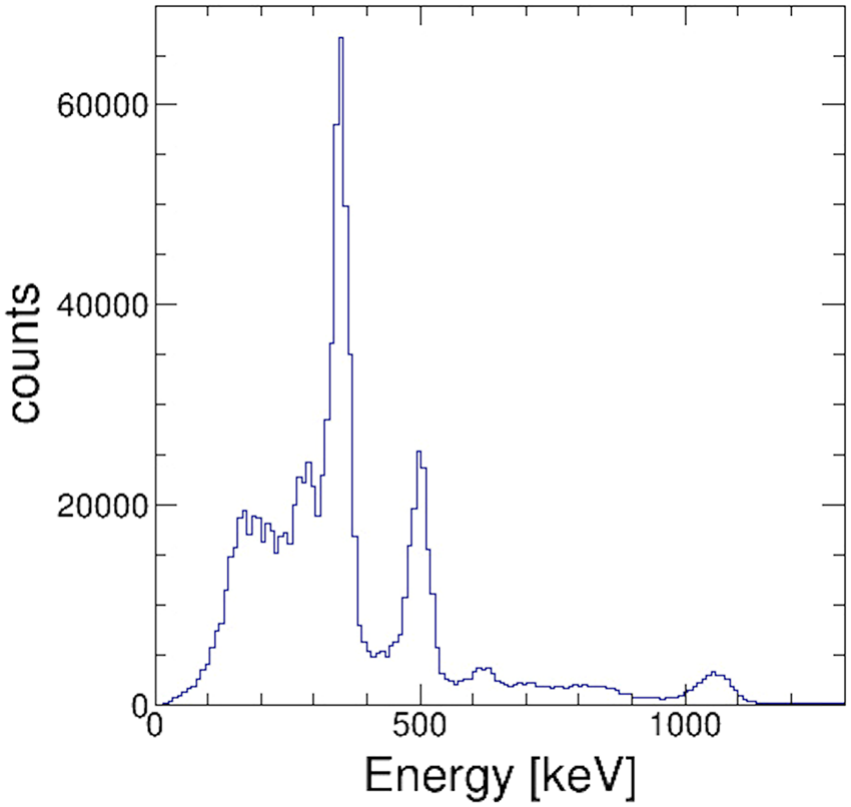
Results of energy spectrum.

**Figure 5:**
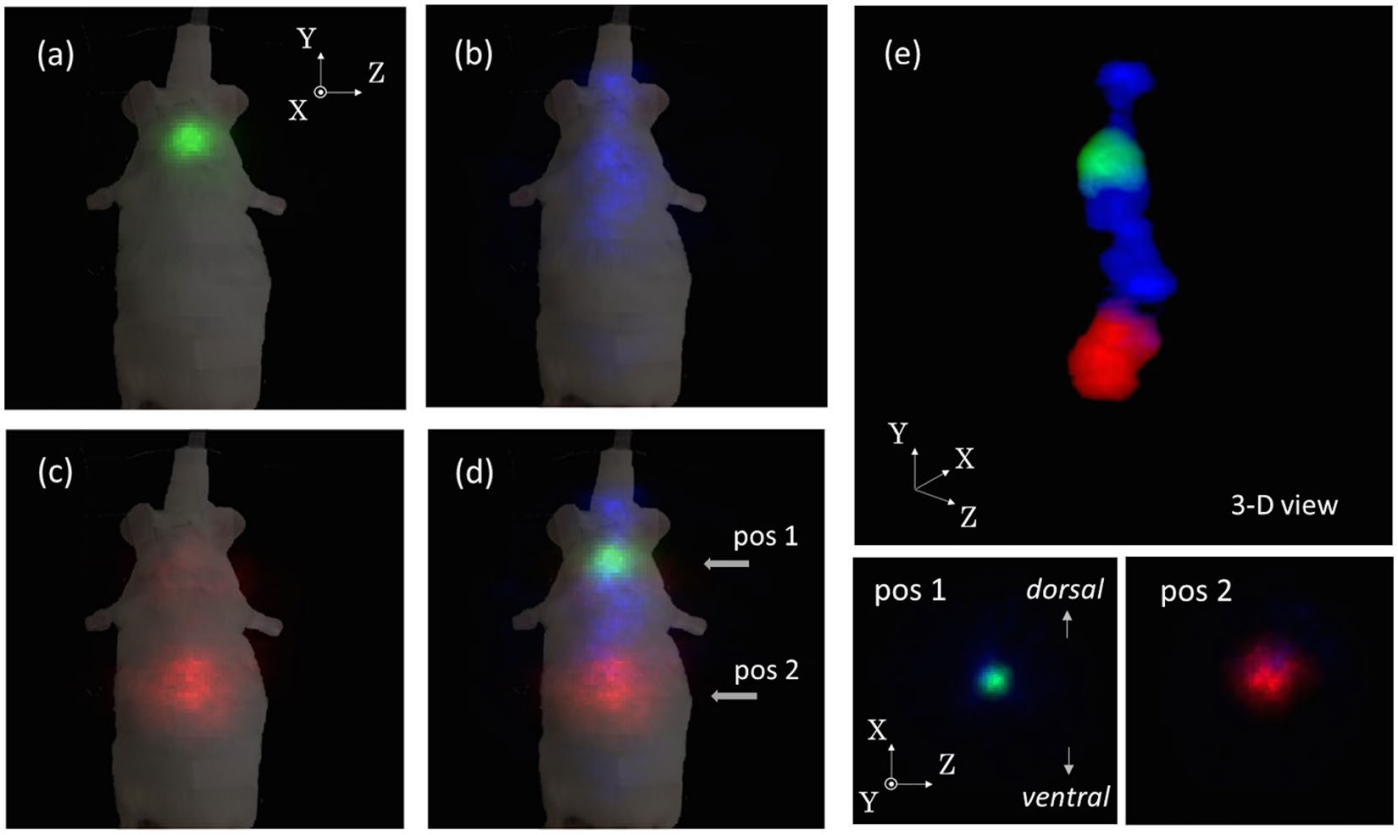
Results of representative 2-D slice in the Z-Y plane of 3-D mouse Imaging. (**a**) Image of ^131^I, (**b**) ^85^Sr, (**c**) ^65^Zn, (**d**) fused images of all three tracers, and (**e**) the 3-D view image and the tomographic images of the fused image in the Z-X plane at the two indicated positions.

In the pre-clinical evaluation of 3-D multi-color imaging, we conducted imaging of a live mouse with three different RI tracers: ^131^I, ^85^Sr, and ^65^Zn. Figure 4 shows the results of the energy spectrum obtained by 10-min measurements from an angle. The three peaks from each tracer −364 keV, 514 keV, and 1116 keV – are clearly seen. Figure 5(a–c) show the results of representative 2-D slices in the Z-Y plane of the 3-D image for each tracer, while Fig. 5(d) shows the fused image of all three tracers. In addition, in order to provide 3-D isotropic imaging capability, Fig. 5(e) shows a 3-D view and tomographic images of the fused image in the Z-X plane at the two indicated positions where the concentration of the radioisotopes is most clearly seen. Note that distribution of ^65^Zn is much broader than ^131^I. These images confirm that the tracers were correctly accumulated in each target organ.

We carried out the quantitative performance evaluation of these imaging results. We compared the relative radioactive intensities by harbesting organs 8 h after the imaging experiment. The accurate experimental RI intensities were measured using a Ge detector. The radioactive intensity of the thyroid, which was measured by the 364 keV peak from ^131^I, was 0.470 MBq: that of the liver, which was measured by the 1116 keV peak from ^65^Zn, was 0.141 MBq. Moreover, we found a substantial uptake of ^65^Zn not only in the liver but also in the lung, heart, intestine, spleen, and pancreas, although the integrated activity was the strongest in the liver. In contrast, such uptake was negligibly small, amounting to less than 10% of target organs, for both ^131^I and ^85^Sr. This can be actually seen in Fig. 5(c) where the concentration of ^65^Zn is much broader compared to ^131^I, such that weak diffuse emission is also seen near the liver, lung, and heart.

To calculate the reconstructed relative intensities, the 3-D ROI (*r* = 7 mm for the thyroid and *r* = 20 mm for the liver) was applied. The intensity for each organ was defined as integral value over the voxels inside the 3-D ROI, considering the effect of the decay probability and decay time from the imaging experiment. Table 2 shows the measured and reconstructed radioactive intensities for both the thyroid and liver. The relative intensity of the reconstructed images corresponded to the real activity with an accuracy of less than 20%.

**Table 2:** Comparison of the intensity of the radioactive tracers.

RI tracer	Evaluated organ	Weights [g]	Measured intensity [MBq] (ratio [a.u.])	Reconstructed intensity ratio [a.u.]
^131^I	thyroid	0.062	0.470 (1.000)	1.000
^65^Zn	liver	1.84	0.141 (0.191)	0.159

These results demonstrate the effectiveness of simultaneous *in vivo* imaging of multiple tracers. Here, we discuss the imaging performance of the Compton camera compared with that of other current modalities such as SPECT and PET. The spatial resolution of the medical Compton camera in this study was slightly worse than that of SPECT and PET for small animals. The principal factor that restricts the spatial resolution of our medical Compton camera is energy uncertainty, although the contribution of position uncertainty becomes dominant above 1 MeV. Detailed simulations provide evidence that the improvement of the energy resolution by 2% at 662 keV makes the resolution of the medical Compton camera nearly equivalent to that of PET. On the othr hand, with regard to the detection efficiency and measurement time, the performance of the medical Compton camera was comparable to SPECT. In addition, this study used and rotated only one Compton camera for multi-angle data acquisition in order to reduce detector costs. However, arranging multiple Compton cameras in a circle can reduce measurement time by approximately 10 min for a similar imaging.

## Methods

### Image reconstruction method

**Figure 6:**
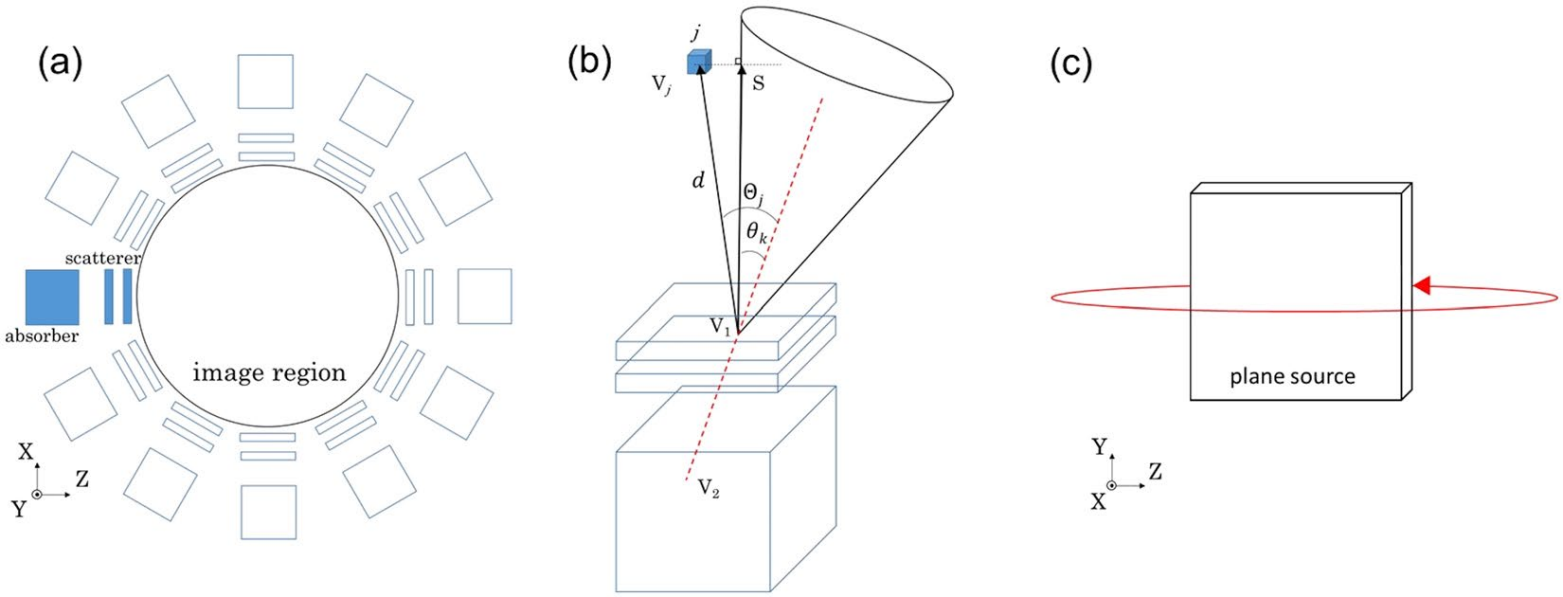
(**a**) Configuration of the multi-angle data acquisition measurement, (**b**) diagram of the 3-D MLEM reconstruction, and (**c**) configuration of the measurement of the plane source.

In order to improve the image anisotropicity which results from the limited-angle problem in typical Compton camera measurements, we propose a multi-angle data acquisition method^19^. In the multi-angle measurement in this study, the Compton camera was rotated around the image region and data was collected from multiple angles as shown in Fig. 6(a). We reconstructed 3-D images using the following algorithm, which is based on list-mode MLEM^20^:

1 $${{\lambda }}_{j}^{n}={{\lambda }}_{j}^{n-1}\,\sum _{i=1}\frac{1}{{s}_{j}^{l}}\frac{{t}_{ij}{v}_{i}}{{\sum }_{k}{t}_{ik}{{\lambda }}_{k}^{n-1}}$$

where *l* is the suffix corresponding to a certain data acquisition angle (*l* = 0, 1, 2, …, 11), ${\lambda }_{j}^{n}$ is the reconstructed image value after *n* ^*th*^ iteration, ${s}_{j}^{l}$ is the probability that a photon emitted from image voxel *j* is detected at a certain acquisition angle *l*, *v*_*i*_ is the probability that an event *i* comes from the image space^21^, and *t*_*ij*_ is the system matrix where a photon emitted from imaging voxel *j* will be measured as event *i*. Figure 6(b) shows the schematic parameters used in the 3-D reconstruction. We incorporated the solid angle of the imaging voxel and the reaction probability of each gamma ray in system matrix *t*_*ij*_:

2 $${t}_{ij}=2\pi (1-\frac{d}{\sqrt{{d}^{2}+{a}^{2}}})\times \exp \,[-\frac{1}{2}{(\frac{|{{\rm{\Theta }}}_{j}|-|{\theta }_{k}|}{\sigma })}^{2}]\times \frac{1}{\sin \,{\theta }_{k}}\times f({E}_{1},{E}_{2},{\theta }_{k},{x}_{s},{x}_{a})$$

3 $$f({E}_{1},{E}_{2},{\theta }_{k},{x}_{s},{x}_{a})=\exp \,\{-{\sigma }_{t}\,({E}_{1}+{E}_{2})\cdot {x}_{s}\}\cdot \frac{d{\sigma }_{C}}{d{\rm{\Omega }}}\cdot \exp \,\{-{\sigma }_{t}({E}_{2})\cdot {x}_{a}\}\cdot {\sigma }_{p,C}$$

where *a* and *d* are the half size of the imaging voxel and the distance between the detector and the voxel, respectively; Θ_*j*_ is the angle between the cone axis and the direction of the interested image voxel *j*; *θ*_*k*_ is the scattering angle calculated by the energy information; and *σ* is the angular resolution of the camera. The first term of equation (2) represents the effect of the solid angle of the voxel seen from the detector; the second term represents a Gaussian distribution from the uncertainty of the cone; and the third term is a weighting factor of each event. The last term *f* is the reaction probability of a photon: $\exp \{-{\sigma }_{t}\,({E}_{1}+{E}_{2})\cdot {x}_{s}\}\,{\rm{and}}\,\exp \{-{\sigma }_{t}\,({E}_{2})\cdot {x}_{a}\}$ are the transportation probabilities of a photon in the scintillator (*x*_*s*_ and *x*_*a*_ are the path length in the scintillator); $\frac{d{\sigma }_{C}}{d{\rm{\Omega }}}$ is the Compton scattering probability of a photon which has the energy *E*_1_ + *E*_2_ in the scattering angle *θ*; and *σ*_*p*,*C*_ is the interaction probability of a photon of energy *E*_2_ by either photoelectric absorption or Compton scattering.

To obtain the 3-D images in both imaging experiments, data was collected for 12 angles, which was equal to a 30° pitch. The image region was defined as 8 × 8 × 8 cm^3^ through the experiments. Our calculation includes both dead-time and decay correctons which correspond to the measurement.

### 3-D imaging of uniform plane source

We utilized a plane source phantom of 30 mm × 30 mm × 3 mm as the uniform plane source, which was filled with ^137^Cs solution. The total intensity of the source was 2 MBq. For the target source, we rotated the medical Compton camera as shown in Fig. 6(c). The data acquisition configuration was 12 angles. Each took 20 min, making the total integration time 4 h.

In this plane source measurement, we evaluated the edge delineation performance and image intensity uniformity as the parameters that defined imaging capability. To evaluate the edge delineation in the Y and Z directions (i.e., the parallel direction to the source plane), the error function was fitted to the 1-D slice of the reconstructed image, and the spatial resolution was calculated from the fitting results. On the other hand, in the X direction, which is equal to the thickness direction, the response *R*(*x*) can be expressed by the following convolution:

4 $$R(x)=\int LSF\,(x-x^{\prime} )\cdot e(x^{\prime} )dx^{\prime} $$

5 $$e(x)=\{\begin{array}{ll}1 & (0\le x\le \mathrm{3)}\\ 0 & (x < 0,x > 3)\end{array}$$

where the line spread function is denoted by *LSF*. Hence, we calculated various responses *R*(*x*) as a function of *σ* of the *LSF*, and the resolution was estimated based on the FWHM value of the *R*(*x*).

Furthermore, in order to evaluate image uniformity, we obtained the 2-D ROI which was determined by eliminating the 2*σ* region of the spatial resolution from the reconstructed edge position. For the ROI, we evaluated the fluctuation of the all voxels based on the averaged value.

**Figure 7:**
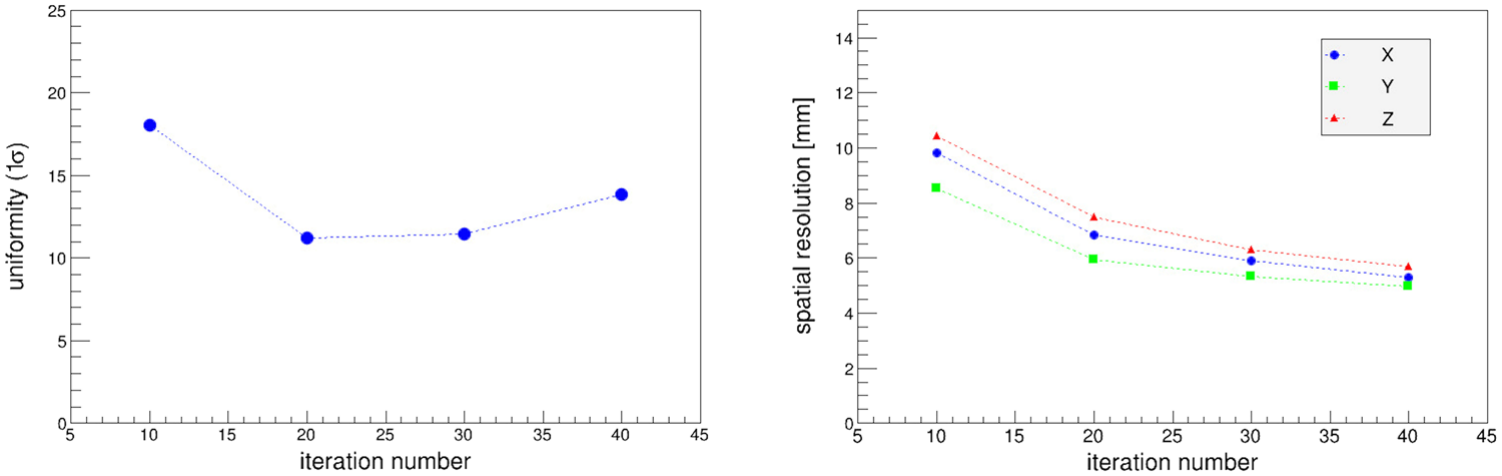
(*Left*) Uniformity (1*σ*) and (*right*) spatial resolution as a function of the number of iteration. The blue, green, and red plots in the *right* figure indicate the spatial resolution in the X, Y, and Z directions, respectively.

To determine of the number of iteration, we optimized in terms of both the uniformity and spatial resolution. Figure 7 (*left*) and (*right*) show the uniformity and spatial resolution as a function of the iteration number, respectively. These results suggest that uniformity had the best value when the iteration number was approximately 20–30. On the other hand, the spatial resolution improved with increasing iteration number in the range of less than 40. This indicates that increasing the number of iterations can improve the spatial resolution, which is close to the values obtained by point source measurements. However, because the amount of data was insufficient, the fluctuations of each voxel stand out and the performance of the uniformity declined. Based on abovementioned reasons, we adopted the iteration number of 30 for the imaging of the plane source.

### 3-D *in vivo* imaging with a living mouse

For the *in vivo* imaging, we studied an eight-week-old male mouse (39.9 g), which fed a low iodine diet for two weeks and was injected via tail vein under inhalation anesthesia (isoflurance/air mixture). Three radioactive tracers were utilized: (1) ^131^I-NaOH (4.0 MBq) was injected two days before the imaging experiment; (2) ^85^SrCl_2_-HCL (1.12 MBq) was injected one day before the imaging experiment; and (3) ^65^ZnCl_2_-saline (0.93 MBq) was injected 1 h before the imaging experiment. The features of these tracers are listed in Table 1. These tracers showed the *in vivo* behavior of accumulation in specific areas: dissociated ^131^I accumulated in the thyroid, ^85^Sr accumulated in the bone, and ^65^Zn accumulated in the liver^22^. The mouse was treated with inhalation anesthesia and placed on the rotation stage in an upright position. The medical Compton camera was rotated around the mouse in the plane which was perpendicular to the body axis of the mouse. The measurement time for each position was 10 min, thus the total integration time was 2 h. All of the animal experiments were approved by the animal ethics committees of Osaka University and were performed according to the institutional guidelines.

**Table 1:** Features of radioactive tracers in mouse imaging.

RI tracer	Energy [keV]	Injected intensity [MBq]	Decay time	Accumulation
^131^I	364	4.0	8 d	thyroid
^85^Sr	514	1.12	65 d	bone
^65^Zn	1116	0.93	244 d	liver
